# Three new species of the sea fan genus *Leptogorgia* (Octocorallia, Gorgoniidae) from the Gulf of California, Mexico

**DOI:** 10.3897/zookeys.1017.50619

**Published:** 2021-02-12

**Authors:** Osvaldo Hernández, Jaime Gómez-Gutiérrez, Carlos Sánchez

**Affiliations:** 1 Departamento de Ciencias Marinas y Costeras, Universidad Autónoma de Baja California Sur, Carretera al sur km 5.5, CP 23080, La Paz, Baja California Sur, Mexico Instituto Politécnico Nacional La Paz Mexico; 2 Departamento de Plancton y Ecología Marina, Centro Interdisciplinario de Ciencias Marinas, Instituto Politécnico Nacional, Av. IPN, s/n, CP 23096, La Paz, Baja California Sur, Mexico Universidad Autónoma de Baja California Sur La Paz Mexico

**Keywords:** Alcyonacea, chromotypes, Cnidaria, gorgonians, microendemism, rocky reef

## Abstract

Three new sea fan species of *Leptogorgia* were discovered during multiple scuba diving expeditions along the Gulf of California coast and islands. *Leptogorgia iridis***sp. nov.** is distributed in the southern region of the gulf (Mexican Province), inhabiting tropical rocky reefs of the Islas Marías Archipelago (Nayarit) and Bahía Banderas (Jalisco). This species has small colonies (< 7 cm height) with at least five clearly distinct chromotypes. *Leptogorgia martirensis***sp. nov.** was found exclusively on the rocky reefs of San Pedro Mártir and San Esteban Islands located in the northern region of the Gulf of California (northern region of Cortez Province). *Leptogorgia enrici***sp. nov.** is distributed from the south to the northern region of the Gulf of California (Cortez Province), inhabiting substrates of rocky reefs, sandy and pebbly sea floors. Comprehensive ecological diving expeditions to identify and classify octocorals in the Mexican Pacific (1995–2019) indicate that *L. iridis***sp. nov.** and *L. martirensis***sp. nov.** are likely to be micro-endemics and *L. enrici***sp. nov.** is endemic to the Gulf of California, which defines their currently known biogeographic distribution ranges.

## Introduction

The family Gorgoniidae Lamouroux, 1812 includes mostly species of three genera in the eastern Pacific: *Pacifigorgia* Bayer, 1951 with anastomosed branching as the main diagnostic character, *Eugorgia* Verrill, 1868 with the presence of double disk capstans and *Leptogorgia* Milne Edward & Haime, 1857; which, in contrast to the previous two genera, does not have a single diagnostic genus morphological feature (Williams et al. 2004; Breedy et al. 2009). Verrill (1868) separated species of the genera *Eugorgia* from *Leptogorgia* because *Eugorgia* species have double disk capstan sclerites. *Leptogorgia* taxonomic classification is based on several morphological characters that might be present or absent in the genera *Pacifigorgia* and or *Eugorgia* (Breedy and Guzman 2002; Breedy et al. 2009). Branching and colony growth patterns and types of sclerites are required morphological diagnostic traits to identify and distinguish among *Leptogorgia* species (Breedy and Guzman 2007). The lack of a single diagnostic taxonomic character in the genus *Leptogorgia* causes uncertainties in the taxonomic classification of species in this highly morphologically diverse genus, which currently includes 103 nominal species and seven species assigned as *nomen dubium* worldwide (Cordeiro et al. 2020). This taxonomical problem is particularly accentuated by multiple species having wide interspecific and intraspecific variability of morphotypes and chromotypes. The molecular evidence strongly suggests that genus *Leptogorgia* has deep genetic divergence among morphologically similar species, with multiple genetic geographically restricted lineages (Poliseno et al. 2017; Soler-Hurtado et al. 2017a, b; Olvera et al. 2018; Silvestri et al. 2019).

There are 30 extant *Leptogorgia* species recorded along the American Pacific coast, with *Leptogorgia waltonae* Olvera, Hernández, Sánchez & Gómez-Gutiérrez, 2018 being the latest species described in the Mexican Pacific (Olvera et al. 2018). Here we describe three new species of the genus *Leptogorgia* discovered in the Gulf of California during extensive ecological diving expeditions to identify and classify octocorals in the Mexican Pacific (1995–2019). Therefore, these three new *Leptogorgia* species increase the total number of nominal *Leptogorgia* species to 33 for the American Pacific and 20 for the Mexican Pacific.

## Materials and methods

Approximately 500 quantitative monitoring transects, each one covering an area of 30 m², were surveyed during extensive annual ecological expeditions located along the peninsular coast and at 25 islands of the Gulf of California (1995–2019), Islas Marías Archipelago (2010 and 2018), Bahía Banderas (2013) and Bahía Magdalena (2013–2014) (Fig. 1). Several octocoral colonies were collected during those monitoring surveys for taxonomic purposes. A total of 35 colonies of *Leptogorgia iridis* sp. nov., 25 colonies of *Leptogorgia martirensis* sp. nov. and 42 colonies of *Leptogorgia enrici* sp. nov. were collected during scuba diving between 2–55 m depths. All specimens were dried or preserved in 96% ethanol. A portion of each colony was macerated in sodium hypochlorite to extract the sclerites, washed several times with distilled water and preserved in 96% ethanol for further microscopic analyses. Sclerites were air-dried and attached to aluminum stubs with double adhesive bands. They were coated with gold using a sputter coater (Polaron E5100) in an argon atmosphere and observed under a Hitachi S-3000 N scanning electron microscopy (SEM) at 20 kV. The sclerite morphological traits were compared with sclerites of fourteen nominal *Leptogorgia* species distributed along the tropical eastern Pacific (Table 1) using original taxonomic descriptions (Breedy and Guzman 2007; Horvath 2011; Breedy et al. 2012; Olvera et al. 2018) and taxonomical analyses from octocoral specimens from the institutional collection of Universidad Autónoma de Baja California Sur (Proyecto Fauna Arrecifal: PFA). Species identification and morphological comparisons among *Leptogorgia* species were assessed following the standard techniques and nomenclature used by Verrill (1868), Breedy and Guzman (2007), Breedy et al. (2009, 2012), and Horvath (2011) (Table 1). We used standard taxonomic terminology and criteria to describe the three new species (Bayer et al. 1983; Calvo and Breedy 2002; Breedy et al. 2009, 2013; Breedy and Guzman 2007, 2013). All the holotypes and paratypes were deposited in the Smithsonian National Museum of Natural History (**NMNH**).

**Figure l.**
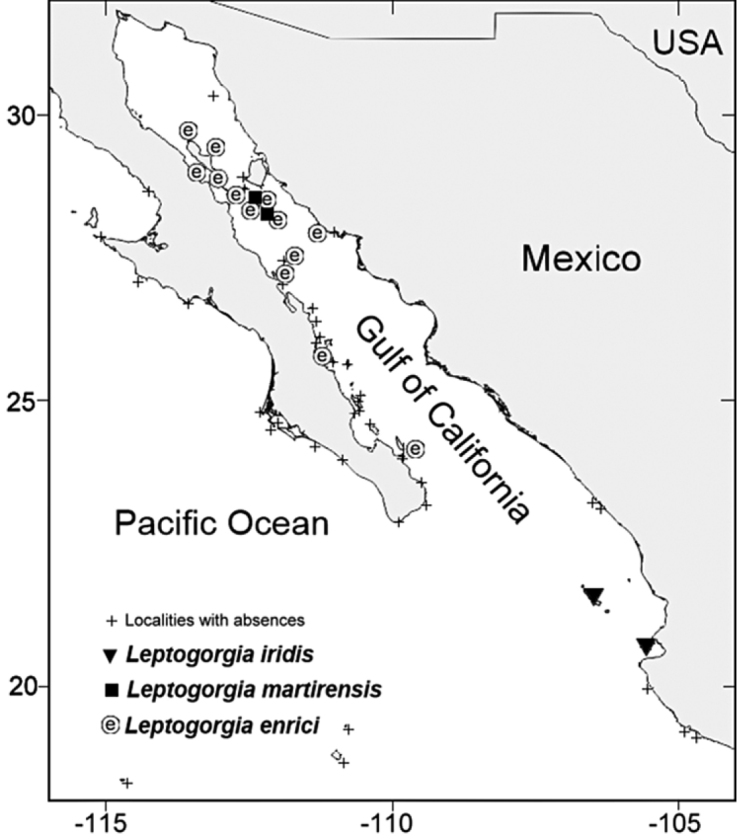
Map of the Gulf of California and Mexican Pacific showing collection localities of the three new species of *Leptogorgia*.

**Table 1.** Comparison of morphological characters of *Leptogorgia iridis* sp. nov., *Leptogorgia martirensis* sp. nov. and *Leptogorgia enrici* sp. nov. with fourteen other *Leptogorgia* nominal species distributed along the Mexican Pacific and Gulf of California of specimens collected from 2002–2016 and from other regions of the Eastern Pacific reported in Breedy and Guzman (2007), Horvath (2011), Breedy et al. (2012), and Soler-Hurtado et al. (2017b). All morphological measurements are given in mm. All the taxonomical characters are based on the holotypes or lectotypes except the color of the colony and the sclerites of several species from the Gulf of California that are based on the characteristics of extra specimens. Branching type: bra-str = branches/strands, di = dichotomous, irr = irregularly, lb = laterally branched, pd = pseudodichotomous, pi = pinnate. Polyp distribution rows: irr = irregular, spar = sparsely. Color: am = ambar, br = brownish, brr = brownish red, hy = hyaline, o = orange, r = red, y = yellow, pi = pink, pu = purple, w = white. Numerically dominant types of sclerites: C = capstan, O = oval, S = spindles.

Species	Colony growth	Branching type	Terminal branches diameter	Polyp distribution rows	Polyp mound elevation	Capstan length	Spindles length	Bent spindles	Crosses	Anthocodial sclerites	Dominant sclerites	Color of colony	Bicolor colonies	Number of solid chromotypes	Bicolor sclerites	Anthocodial sclerites color	Color ring
***L. iridis* sp. nov.**	planar	lb	1–2	2	slightly raised	0.06	0.07	no	0.05 × 0.05	rod	C	pu/y,pu, r, w, y	yes	5	yes	pu, r, w, y	no
***L. martirensis* sp. nov**	bushy	lb	2	1–2	prominent	0.05	no	no	no	rod	C	y, p, br	no	3	yes	o, r, w	no
***L. enrici* sp. nov.**	planar	lb	1.5	1, irr	no	0.06	0.11	yes	0.06 × 0.06	rod	C	o, pu, y, w, y/pu	yes	4	no	o, y, pu	no
*L. alba*	flabellate	irr/di	–	2	slightly raised, flat	0.06	0.18	yes	–	rod	S	w	no	1	no	no	no
*L. chilensis*	lank, bushy	irr/di	2.8	spar	flat	0.08	0.12	no	0.06 × 0.06	Rod, biscuit	C-S	o	no	1	no	o	no
*L. clavata*	–	pi	–	irr	slightly raised	0.075	0.10	no	–	rod	C	r	no		no	pi	no
*L. cuspidata*	bushy	irr/pd	3.25–4	crowd	–	0.09	0.13	yes	–	rod	C	pu/y	yes	1	yes	y	yes
*L. diffusa*	bushy, arborescent	lb	3	1–2	prominent	0.09	0.15	yes	–	rod	S	o	no	1	no	o	no
*L. ena*	cluster	lb	2–3	irr	slightly raised	0.086	0.108	no	0.05 × 0.07	rod-platelet	C	pu, y, pu/y	yes	3	yes	pu, y	no
*L. exigua*	bushy	irr/pi	3–4	crowd	slightly raised	0.10	0.13	no	–	–	C	brr/y	no	1	yes	–	no
*L. filicrispa*	strands	bra-str	0.5–1	2	prominent	0.08	0.11	no	–	rod	–	pi	no	1	no	pi	no
*L. flexilis*	bushy	irr/di	1.0–1.5	4–5	flat	0.09	0.09	no	–	rod	C	r/br	no	1	yes	r	no
*L. labiata*	flabellate	irr/pi	2	4	prominent	0.08	0.1	no	–	rod	C	pi/y	yes	1	yes	y	yes
*L. laxa*	planar	irr/di	1.0–1.5	2	slightly raised	0.08	0.18	no	–	rod	–	w	no	1	no	no	no
*L. manabiensis*	planar	irr/pi	1.9	spar	slightly raised	0.08	0.14	yes	–	rod	S	pi	no	1	no	no, hy	no
*L. pumila*	bushy	irr/pi	2, 3	2, spar	raised	0.1	0.15	yes	0.08 × 0.06	rod	–	pu, pi	no	1	no	am	no
*L. rigida*	bushy, arborescent	pi	2–3	3–4	slightly raised	0.08	0.12	no	0.04 × 0.04	rod	C, O	pu	no	1	no	pi	no

## Systematics

### Class Anthozoa Ehrenberg, 1834

**Subclass Octocorallia Haeckel, 1866**

**Order Alcyonacea Lamouroux, 1812**

**Suborder Holaxonia Studer, 1887**

**Family Gorgoniidae Lamouroux, 1812**

#### Genus *Leptogorgia* Milne Edward & Haime, 1857

##### Leptogorgia iridis sp. nov.

Taxon classificationAnimaliaAlcyonaceaGorgoniidae

C60F5277-D18F-5D2C-9030-2E257009C652

http://zoobank.org/38587E54-6E24-4949-BCF5-61FBB2982023

Figures 3 , 8A, B

###### Material examined.

***Holotype*.**NMNH-1638551: dry María Magdalena Island (south west rocky point), Islas Marías Archipelago, Nayarit, Mexico (21°25.267'N, -106°24.900'W), 10 m depth, 15 November 2010, collector Carlos Sánchez. ***Paratypes*.**NMNH-1638552: dry María Madre Island (southern rocky point), Islas Marías Archipelago, Nayarit, Mexico (21°32.391'N, -106°31.877'W), 8 m depth, 18 November 2010, collector Carlos Sánchez. NMNH-1638553: dry María Madre Island (southwest rocky point, Islas Marías Archipelago, Nayarit, Mexico (21°32.391'N, -106°31.877'W), 8 m depth, 18 November 2010, two colonies connected by single holdfast, collector Carlos Sánchez. NMNH-1638554: dry, María Madre Island (southwest rocky point), Islas Marías Archipelago, Nayarit, Mexico (21°25.267'N, -106°24.900'W), 10 m depth, 15 November 2010, collector Carlos Sánchez. NMNH-1638555: dry colony from El Faro de Cabo Corrientes, Bahía Banderas, Jalisco, Mexico (20°24.553'N, -105°41.708'W), 2013, collector Carlos Sánchez.

###### Type locality.

Islas Marías Archipelago is located in the southern region of the Gulf of California, Mexico (21°25.267'N, -106°24.900'W) near the continental shelf-break about 158 km southwest of Mazatlán, Sinaloa and 106 km northwest of Bahía Banderas, Nayarit (Fig. 1).

###### Holotype colony description.

**Figure 2.**
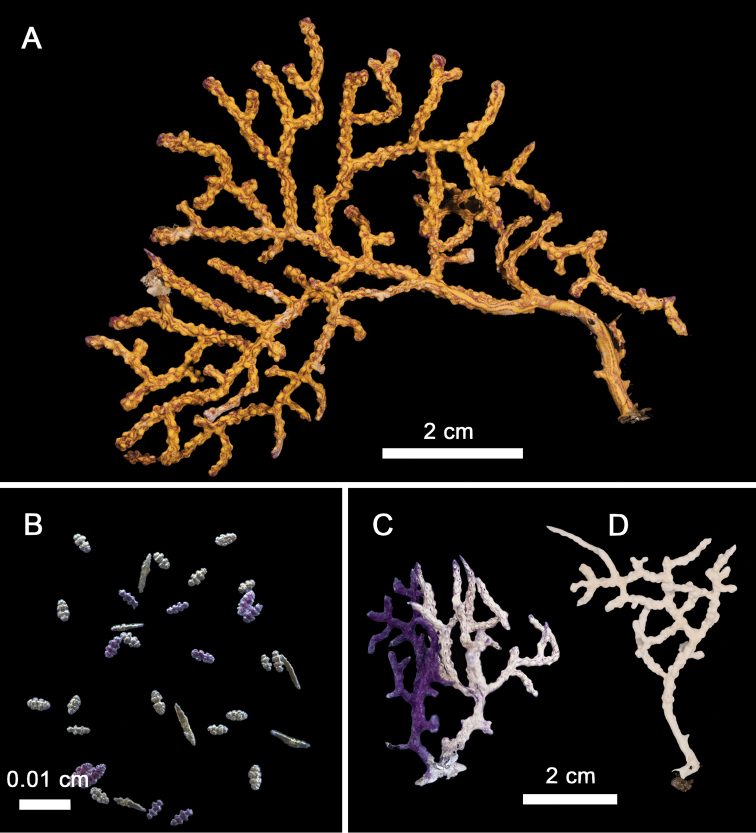
*Leptogorgia iridis* sp. nov. **A** holotype NMNH-1638551 **B** holotype anthocodial and coenenchymal sclerites **C** paratype NMNH-1638553, two colonies with different color connected with a single holdfast **D** paratype NMNH-1638554, monochromatic white chromotype.

Colony shows lateral branching and planar growth of 7 cm height and 8.1 cm width. Holdfast is 5 mm diameter and arises the main steam 2.1 cm length and 2 mm diameter, subdividing into two main branches (Fig. 2A). The longer branch grows up to 2.8 cm length and 2 mm diameter before dividing into secondary and further branches 1–2 mm in diameter. The smaller branches are 4 mm length and 1 mm diameter before subdividing and growing downward. The main stem shows three alternating and broken pinnula with a brownish nude axis of 0.5 mm diameter. Secondary and terminal twigs have blunt tips arising at 45° angles and of > 2 mm diameter. The entire colony is yellow and deep purple, forming longitudinal bicolor striped patterns along the branches from the base to the tips of the colony (Fig. 2A). Polyp mounds are oval 1.0 × 0.5 mm, slightly raised by 1 mm with polyp rings, arranged in two rows along with the entire colony, except on the lower half of the stem.

###### Holotype sclerites.

**Figure 3.**
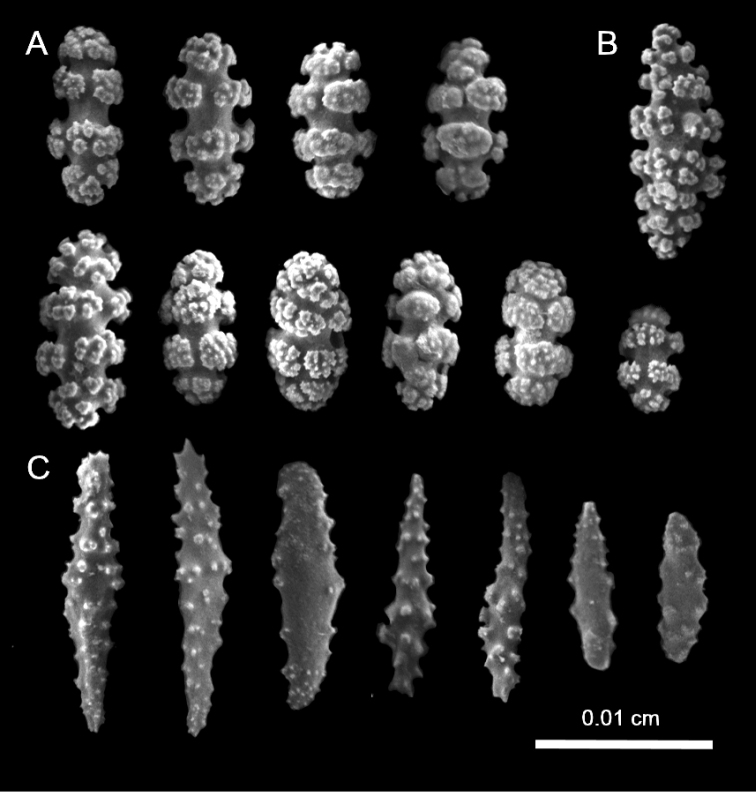
*Leptogorgia iridis* sp. nov., Scanning Electron Microscopy images of coenenchymal sclerites from the holotype NMNH-1638551 **A** capstans **B** spindle **C** anthocodial rod sclerites.

Coenenchymal sclerites of *Leptogorgia iridis* sp. nov. holotype are mostly bright yellow or purple and few of them are bicolor or white (Fig. 2B). Dominant sclerites are capstans (0.06 mm length and 0.04 mm width) (Fig. 3A). Spindles are scarce (0.07 mm length and 0.03 mm width), slightly tuberculate, of white color with pale orange in the middle (Fig. 3B). Crosses measure up to 0.05 mm length and 0.05 mm width. Anthocodial sclerites are long rods of < 0.1 mm length and 0.02 mm width with acute ends and lobed margins (Fig. 3C).

###### Morphological variations.

*Leptogorgia iridis* sp. nov. paratypes differ from the holotype in branch diameter and coloration. The morphotypes have a wide range of colorations due to the different proportion of sclerite colors and coenenchymal sclerite arrangement (Figs 2A–D, 8A, B). There are four solid sclerite colorations: yellow, red, purple, and white or with a gradient of colorations among them, including bicolor chromotypes. A colony may have one or two sclerite colors, but their proportion and combination may create different patterns in the colony’s appearance. The holotype has yellow and purple sclerites with a longitudinal color arrangement giving the colony a bicolor (yellow and purple) appearance (Fig. 2A, B). The paratype NMNH-1638553 also has a mixing of sclerites, one colony has the major sclerite proportion of purple compared to white, and the other colony has a major proportion of white compared to purple sclerites, and both colonies have a scrambled sclerite arrangement giving the colonies their coloration (Fig. 2C). However, in several specimens, such as paratype NMNH-1638554, the colony and sclerite coloration is white (Fig. 2D). Colony growth forms of *L. iridis* sp. nov. have relatively low variability. The only different morphotype, so far collected exclusively at Bahía Banderas, Jalisco, were colonies with similar coloration patterns to the holotype, but with relatively thicker branches (up to 4 mm diameter).

###### Diagnosis.

Purple and red *Leptogorgia iridis* sp. nov. have quite similar colony shapes. Both *L. iridis* sp. nov. chromotypes resemble the color of *Leptogorgia obscura* Bielschowsky, 1929 and *Leptogorgia parva* Bielschowsky, 1929. However, *L. obscura* has small anthocodial rods with blunt ends and *L. parva* has anthocodial rods with conspicuous lobed margins, which are absent in *L. iridis* sp. nov. Additionally, *L. obscura* and *L. parva* have only one known chromotype, and their terminal branches have acutely pointed ends. In contrast, *L. iridis* sp. nov., has long anthocodial rods with acute ends and no lobed margins, showing up to five solid colony chromotypes and terminal branches with blunt ends.

###### Habitat and distribution.

The distribution of *Leptogorgia iridis* sp. nov. covers part of the Central Tropical Mexican Pacific (Mexican Province in Brusca and Wallerstein 1979 and Hasting 2000) from Bahía Banderas, Jalisco to Islas Marías Archipelago Nayarit, Mexico (Fig. 2). *Leptogorgia iridis* sp. nov. grows on shallow rocky reefs < 20 m depth. Purple colonies were mostly observed in shallow waters < 5 m depth, the bicolor colonies mostly at 7–8 m depth, and yellow colonies mostly observed at 10–20 m depth. *Leptogorgia iridis* sp. nov. shares habitat with *Leptogorgia ena* Breedy, Abeytia & Guzman, 2012, *Leptogorgia rigida* Verrill, 1864, *Leptogorgia alba* (Duchassaing & Michelotti, 1864), *Pacifigorgia arenata* (Valenciennes, 1846), *Pacifigorgia agassizii* (Verrill, 1864), *Pacifigorgia media* (Verrill, 1864), *Pacifigorgia stenobrochis* (Valenciennes, 1846), *Muricea austera* Verrill, 1869, and *Heterogorgia papillosa* Verrill, 1870.

###### Etymology.

*Leptogorgia iridis* sp. nov. is named from the Latin word *iridis*, which means “rainbow” due to the large number of chromotypes observed in the colonies. Large numbers of chromotypes are one of the main diagnostic characteristics of this novel tropical species.

##### Leptogorgia martirensis sp. nov.

Taxon classificationAnimaliaAlcyonaceaGorgoniidae

034057AD-1509-558C-8B9F-55DF48A7F3EB

http://zoobank.org/2F4C8356-9EF8-4772-A607-CBCC74032DB8

**Figure 4.**
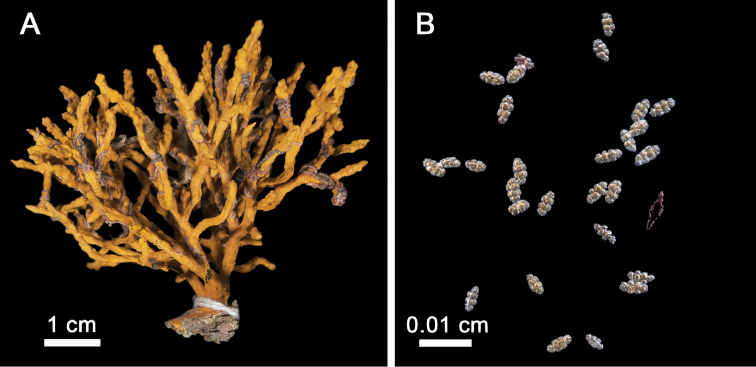
*Leptogorgia martirensis* sp. nov. **A** holotype NMNH-1638556 **B** anthocodial and coenenchymal sclerites.

**Figure 5.**
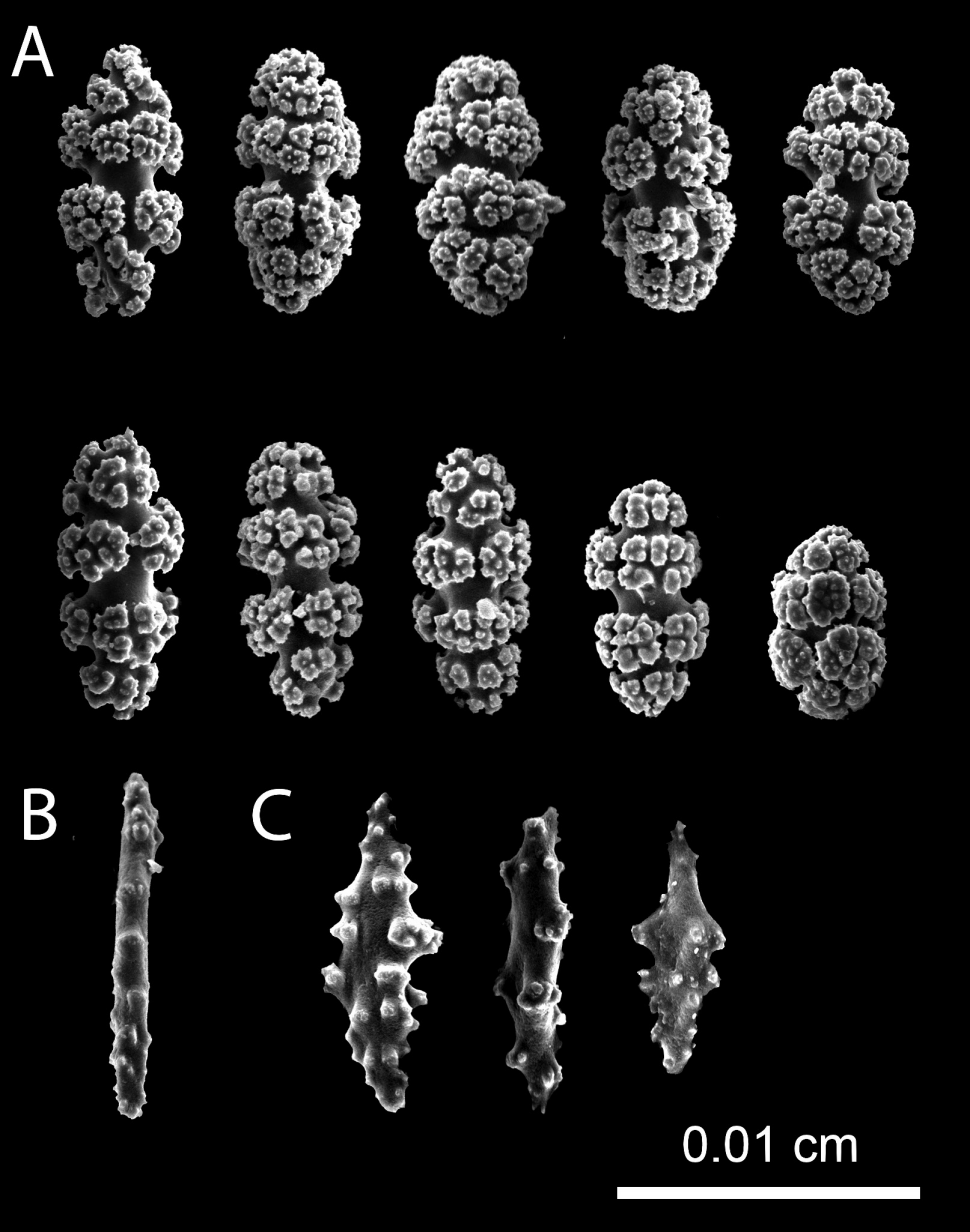
*Leptogorgia martirensis* sp. nov., Scanning Electron Microscopy images of coenenchymal sclerites from the holotype NMNH-1638556 **A** capstans **B** rod lateral view **C** anthocodial rod sclerites.

Figures 1 , 4 , 5 , 8C, D

###### Material examined.

***Holotype*.**NMNH-1638556: dry, Cueva Refugio, San Pedro Mártir Island, Sonora, Mexico (28°22.297'N, -112°19.040'W), 1 m depth, 16 July 2010, collector Carlos Sánchez. ***Paratypes*.**NMNH-1638557: dry, Cueva Refugio, San Pedro Mártir Island, Sonora, Mexico (28°22.297'N, -112°19.040'W), 1 m depth, 16 July 2010, collector Carlos Sánchez; NMNH-1638558: dry, Cueva Refugio, San Pedro Mártir Island, Sonora, Mexico (28°22.297'N, -112°19.040'W), 1 m depth, July 16, 2010, collector Carlos Sánchez; NMNH-1638559: San Pedro Mártir Island, Sonora, Mexico (28°22.818'N, -112°18.4422'W), 20 m depth, 16 July 2010, collector Carlos Sánchez.

###### Type locality.

Cueva Refugio, San Pedro Mártir Island, Sonora, Mexico is one of the furthest offshore islands in the Gulf of California (part of midriff islands at the upper Gulf) where volcanic rocky reefs predominate. San Pedro Mártir Island is a UNESCO “Islas del Golfo de California” Biosphere Reserve (Fig. 1).

###### Holotype colony description.

A yellow colony with bushy and dense growth with multiple and irregular brownish lines (Fig. 4A). The colony is 6.1 cm in length and 8.1 cm in width. The holdfast is irregular, 14 mm × 11 mm from which the short main stem rises (2 mm length, 4 mm diameter). The colony has four main branches up to 11 mm length and 3 mm diameter. The main branches subdivide into multiple secondary branches (up to 31 mm length, 2 mm diameter). Terminal twigs are flat and short (12 mm length, 2 mm width) with acute ends. The general pattern of upward ramification is lateral at 45° angle. Polyp mounds are oval and prominent, forming one or two rows at each side of the branches with 0.5 mm height, 2 mm length, and 1 mm width with elongated calyces. The colony has several specimens of unidentified dried ophiuroids (< 2 mm oral disc diameter) attached to the branches (Fig. 4A).

###### Holotype sclerites.

The coenenchymal sclerites are exclusively capstans (Figs 4B, 5A). There is no evidence of other types of sclerites being present in any other section of the colony. The capstans reach 0.05 mm long and 0.03 mm wide (Fig. 5A), their color is pale yellow, pink, red or bicolor yellow-red, but the predominant color is pale yellow (90%). The anthocodial sclerites are lobed rods with acute or blunt ends up to 0.1 mm length and 0.03 mm width in the center (Fig. 5B, C). They are bicolor white-red, red, orange or white. The red chromotype is predominant (70% of observed colonies) (Fig. 4B).

###### Morphological variations.

*Leptogorgia martirensis* sp. nov. colonies show three chromotypes: purple, yellow and brown (Figs 4A, 8C, D). The colony color depends on the proportion of the dominant color of the coenenchymal sclerites, but in a few cases the colonies show a brown chromotype when the color proportion of sclerites is approximately 50% purple and 50% yellow.

###### Diagnosis.

The colony growth, size and polyp mounds of *Leptogorgia martirensis* sp. nov. are similar to those of *Leptogorgia aequatorialis* Bielschowsky, 1929, *Leptogorgia obscura* and *Leptogorgia parva*. However, these three species each have only one chromotype (purple, pink, and orange, respectively), and all these species have spindles in their coenenchyme up to 0.1 mm length, while *L. martirensis* sp. nov. has three chromotypes and no spindles in the coenenchyme.

###### Habitat and distribution.

The micro-endemic *Leptogorgia martirensis* sp. nov. is only recorded in rocky shallow waters (up to 10 m depth), and low abundance, at San Pedro Mártir and San Esteban Islands, Sonora. The islands are located in the northern Gulf of California (as part of the Cortez Province according to Brusca and Wallerstein 1979, Hasting 2000), and are the most isolated islands in the gulf (Fig. 1). The Cortez Province is associated with the lowest winter sea superficial temperature (SST 15 °C), the widest annual range of SST (15–30 °C), high marine productivity, and harbor a unique macroinvertebrate community, dominated by endemic octocorals of the genus *Muricea* (Ulate et al. 2016). *Leptogorgia martirensis* sp. nov. shares its habitat with *Muricea austera* Verrill, 1869, *Muricea plantaginea* (Valenciennes, 1846), *Muricea* spp., *Psammogorgia teres* Verrill, 1868, and *Eugorgia excelsa* Verrill, 1868.

###### Etymology.

*Leptogorgia martirensis* sp. nov. takes its name from the collection site San Pedro Mártir Island.

##### Leptogorgia enrici sp. nov.

Taxon classificationAnimaliaAlcyonaceaGorgoniidae

828E66EC-DDB4-5A41-9B60-6B98EFEDF3F9

http://zoobank.org/A3DE39AC-113D-436E-834B-4084F5B6F44F

**Figure 6.**
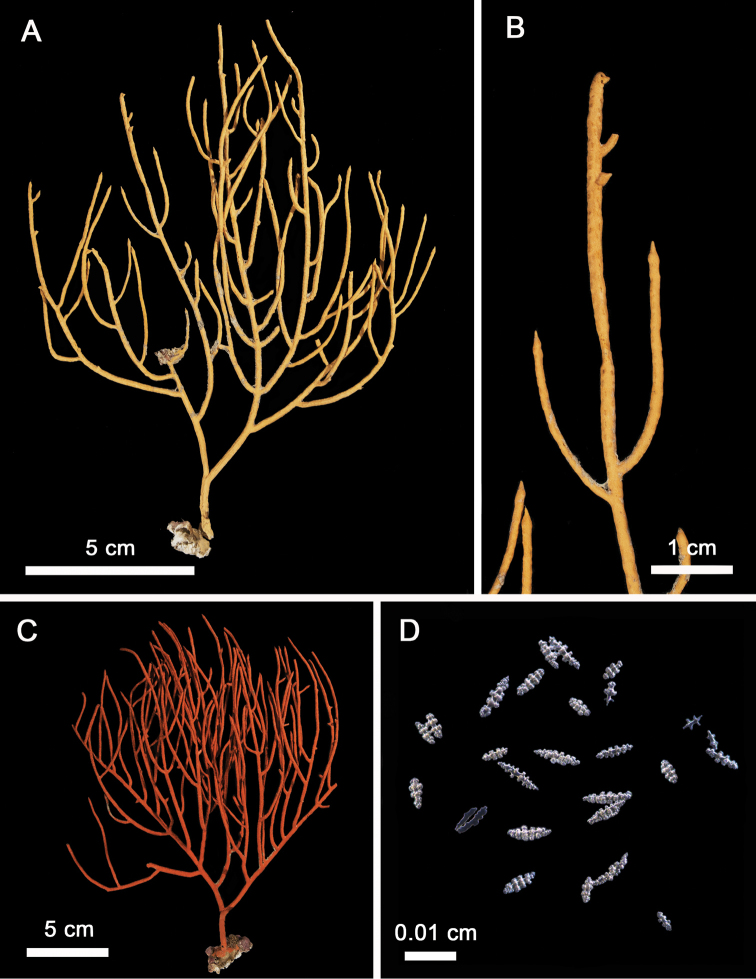
*Leptogorgia enrici* sp. nov. **A** holotype NMNH-1638560 **B** close up of terminal twig **C** orange chromotype colony **D** anthocodial and coenenchymal sclerites.

**Figure 7.**
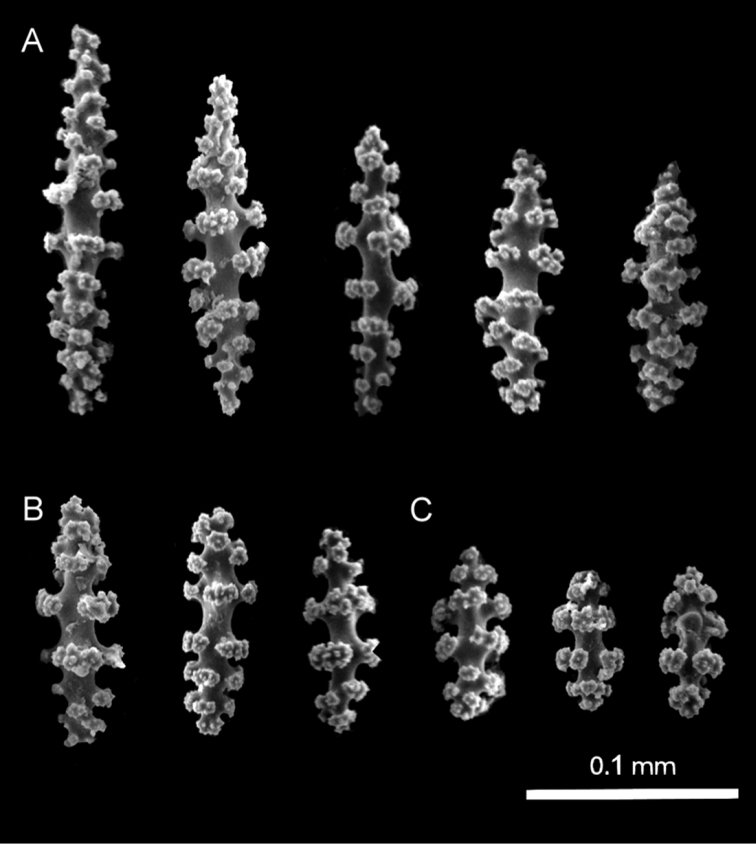
*Leptogorgia enrici* sp. nov., Scanning Electron Microscopy images of coenenchymal sclerites from the holotype NMNH-1638560 **A** acute spindles **B** dull spindles **C** capstans.

**Figure 8.**
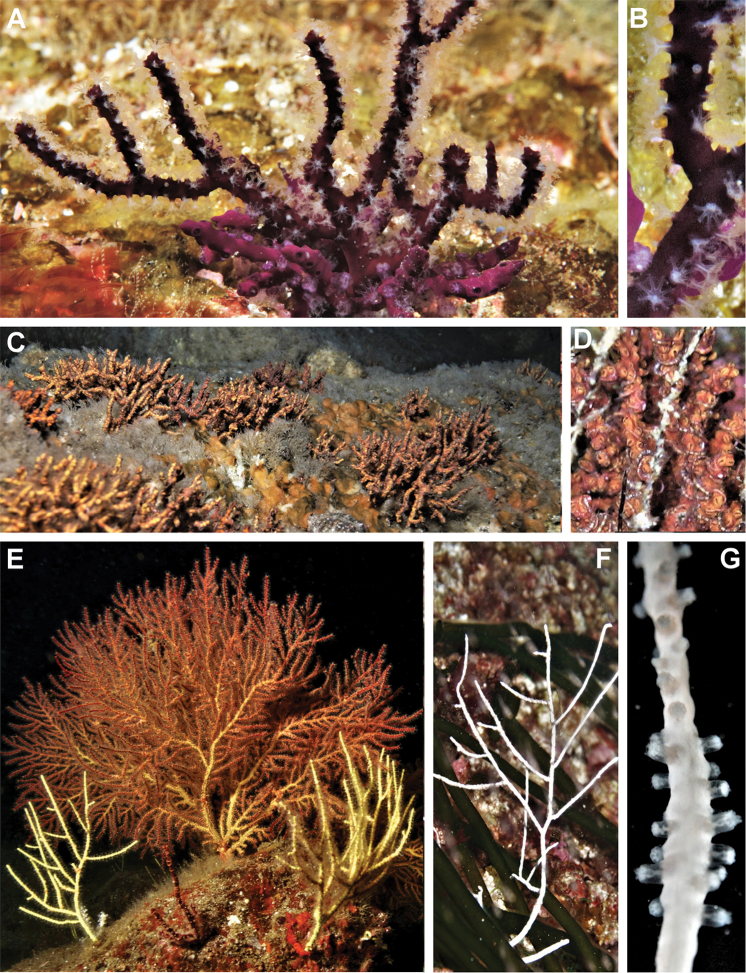
Three new species of sea fans, colonies *in situ*, underwater images **A***Leptogorgia iridis* sp. nov., San Juanito Island, Piedra El Morro, Islas Marías Archipelago, 10 m depth, 23 November 2010, a deep purple colony, at the base a small red wine colony of *Leptogorgia ena***B***Leptogorgia iridis* sp. nov., polyps close up **C***Leptogorgia martirensis* sp. nov., Cueva Refugio, San Pedro Mártir Island, Sonora, 2–3 m depth, 16 July 2010 into the cave several small colonies **D***Leptogorgia martirensis* sp. nov., colony close up **E***Leptogorgia enrici* sp. nov., Los Choros, BC, 25 m depth, 10 July 2009, two yellow colonies, a large colony of *Eugorgia multifida* in the background **F***Leptogorgia enrici* sp. nov., El Bajo Sur, Cerralvo Island, BCS, 30 m depth, 23 June 2006, white colony **G***Leptogorgia enrici* sp. nov., polyps close up. Photographs by Carlos Sánchez.

Figures 1 , 6 , 7 , 8E–G

###### Material examined.

***Holotype*.**NMNH-1638560: dry, San Esteban Island (northwest rocky point), Sonora, Mexico (28°43.564'N, -112°36.799'W), 24 m depth, *in situ* temperature 19 °C, 01 November 1999, collector Carlos Sánchez. ***Paratypes*.**NMNH-1638561: dry, San Esteban Island (northwest rocky point), Sonora, Mexico (28°43.564'N, -112°36.799'W), 24 m depth, *in situ* temperature 19 °C, 01 November 1999, collector Carlos Sánchez; NMNH-1638562: dry, San Esteban Island (northwest rocky point), Sonora, Mexico (28°43.564'N, -112°36.799'W), 24 m depth, *in situ* temperature 19 °C, 03 November 1999, collector Carlos Sánchez; NMNH-1638563: dry, San Pedro Nolasco Island (south rocky point), Sonora, Mexico (27°57.094'N, -111°22.001'W), 30 m depth, 20 October 1999, collector Carlos Sánchez.

###### Type locality.

San Esteban Island is part of the midriff islands at the upper Gulf of California, and is the 15^th^ largest island in Mexico by area (40 km²), and has predominantly volcanic rocky reefs. San Esteban Island is a UNESCO “Islas del Golfo de California” Biosphere Reserve (Fig. 1)

###### Holotype colony description.

A bright yellow colony with planar growth and lateral branching (Fig. 6A, B). The colony is 15.3 cm high and 115 cm wide. The colony has a 9 mm diameter holdfast attached to a rock of small size (14 mm × 11 mm) of biogenic origin from which emerges the main stem of 15 mm length and 2 mm diameter. The stem has longitudinal grooves. From the stem arise two main branches: one of 35 mm length and 2 mm diameter and the other of 117 mm length and 2 mm diameter. From these branches arise multiple secondary laterally growing branches. The terminal branches measure 20–30 mm long, 1.5 mm diameter, and have sharp points (Fig. 6B). The polyp mounds are oval of 1 mm length and 0.5 mm width. Mounds are slightly evident with no elevation and are arranged irregularly or in rows on each side of all branches but not the stem.

###### Holotype sclerites.

The dominant type of sclerites is capstans of 0.06 mm length and 0.03 mm width (Fig. 7C). There are abundant long spindles up to 0.11 mm long and 0.02 mm thick, which may or may not be slightly curved at the tips (Fig. 7A, B). Crosses are unusual, of 0.06 mm × 0.06 mm diameter (not shown). Anthocodial sclerites are mostly small yellow rods of up to 0.05 mm length and 0.01 mm width, these anthocodial sclerites have smooth edges and blunt tips (Fig. 6D). Long rods are also present, but in considerably low proportion.

###### Morphological variations.

*Leptogorgia enrici* sp. nov. has arborescent and planar forms of colony growth. The planar colony is the more common morphotype. *Leptogorgia enrici* sp. nov. has four solid colony colorations: yellow (Figs 6A, B, 8E), orange (Fig. 6C), purple, and white (Fig. 8F, G) plus a rare bicolor colony (yellow with purple rings around the calices). The sclerites of the coenenchyme always have the same coloration as the colony.

###### Diagnosis.

The purple chromotype of *Leptogorgia enrici* sp. nov. is morphologically similar to the thin and planar morphotype of *Leptogorgia rigida*; however, both species differ completely in the form of their sclerites. The coenenchyme sclerites of *L. rigida* consist mainly of robust capstans with short waists, double heads and spheres (absent in *L. enrici* sp. nov.), while the sclerites of *L. enrici* sp. nov. are mainly thin capstans and long and spindle sclerites; spindles are absent in *L. rigida*. These two species are distributed in different habitats: *L. rigida* in shallow areas (<10 m depth) attached to rocky reefs, typically inhabiting areas with strong currents or wave action and even in the cracks of rocks, while *L. enrici* sp. nov. is found in rocky reefs, sandy or pebble beds at depths usually < 20 m depth. The morphology of *L. enrici* sp. nov. is similar in the type of branching and colony color to *Leptogorgia chilensis* (Verrill, 1868) and *Leptogorgia flexilis* (Verrill, 1868). However, these three species are distinct because *L. enrici* sp. nov. has colonies with planar growth and four solid chromotypes (yellow, orange, purple and white) and has many long spindles. *Leptogorgia chilensis* and *L. flexilis* show arborescent growth typically with branches very close to each other. Each species has a single colony chromotype (*L. chilensis* is orange and *L. flexilis* is red) and spindle sclerites are present in low proportions, with blunt tips rather than the long spindles with pointed tips observed in *L. enrici* sp. nov. The long and acute spindles in *L. enrici* sp. nov., are only comparable in size to the spindles of *Leptogorgia alba* and *Leptogorgia manabiensis* Soler-Hurtado, Megina, Machordom & López-González, 2017 (Soler-Hurtado et al. 2017b). However, these long spindles are the dominant type in *L. alba* and *L. manabiensis*, they are broad with acute ends and crowded tubercles. The dominant type of sclerites of *L. enrici* sp. nov. are capstans, the spindles are thin with blunt tips and with sparse tubercles. The anthocodial rods of *L. alba* and *L. manabiensis* are flat, long and have scalloped margins; while the anthocodial rods of *L. enrici* sp. nov. are mostly short with lobed margins and blunt tips.

###### Habitat and distribution.

*Leptogorgia enrici* sp. nov. is endemic to the Gulf of California (Cortez Province according to the biogeographic regions of Brusca and Wallerstein 1979 and Hasting 2000). *Leptogorgia enrici* sp. nov.’s highest densities are concentrated at the northern Gulf of California (northern Cortez sub-province) (Fig. 1), associated with the lowest winter sea surface temperature (SST, 15 °C), the widest annual range of SST (15–30 °C), and high marine productivity (Ulate et al. 2016). *Leptogorgia enrici* sp. nov. inhabits substrates of rocky reefs, or pebbly and shell seafloor habitats surrounded by sand, in shallow waters (5–40 m depth), but most frequently between 20–40 m. *Leptogorgia enrici* sp. nov. may also be distributed in deeper waters.

*Leptogorgia enrici* sp. nov. occurs in low densities scattered on the reefs (< 1 colony 100 m^2^) and never clustered in several colonies. Marine ecological censuses carried out during 2009, 2010 and 2018 showed *L. enrici* sp. nov. is distributed at the Mid-Rift Archipelago of the Gulf of California (Ángel de la Guarda, Partida, Salsipuedes, Las Ánimas, San Lorenzo, San Esteban, San Pedro Mártir, Tortuga and San Marcos) and at the coast of Baja California peninsula (Los Choros). *Leptogorgia enrici* sp. nov. has been collected with scuba at 40 m in the central and southern Gulf of California (Isla Danzante and Isla Cerralvo). *Leptogorgia enrici* sp. nov. shares its habitat with *Muricea* spp., *Muricea plantaginea* (Valenciennes, 1846), *Muricea austera* Verrill, 1869, *Muricea fruticosa* Verrill, 1869, *Eugorgia aurantiaca* (Horn, 1861), *Psammogorgia teres* Verrill, 1868, and *Heterogorgia papillosa* Verrill, 1870.

###### Etymology.

*Leptogorgia enrici* sp. nov. is named in honor of Dr. Enric Sala, a National Geographic Explorer-in-Residence actively engaged in the exploration, research, and science communication to advance ocean conservation. Enric Sala is a passionate enthusiast of marine life and the conservation of Mexican seas who actively collaborates to generate marine biodiversity knowledge. He founded and leads the National Geographic’s Pristine Seas project that has conducted 30 expeditions in the world, creating 22 no-take large marine reserve (~5 million km^2^ of no-fishing zones).

## Discussion

We discovered three new species of the genus *Leptogorgia* in the Gulf of California, adding biodiversity information on the Eastern Pacific Ocean. Although sea fans are the most abundant benthic macroinvertebrates in the rocky reefs of the Gulf of California (Ulate et al. 2016), their taxonomic identities and geographic delimitations in the Gulf of California have been historically poorly studied (Hernández 2014). Ten out the 30 nominal *Leptogorgia* species known for the Eastern Pacific have been described between 2000–2018 (Bayer 2000; Horvath 2011; Breedy et al. 2012; Soler-Hurtado et al. 2017b; Olvera et al. 2018). Bayer (1981) estimated that about 50% of the sea fan species distributed in the Eastern Pacific were then unknown. This study helps to lessen the knowledge gap of total species for *Leptogorgia* via the description of these three new species.

The description of *Leptogorgia iridis* sp. nov., *Leptogorgia martirensis* sp. nov. and *Leptogorgia enrici* sp. nov. increases the number of nominal *Leptogorgia* species currently known in the Mexican Pacific to 20 (Verrill 1868; Bayer 1981; Breedy and Guzman 2005, 2007; Hovart 2011; Breedy et al. 2012; Olvera et al. 2018). Even though Linnean morphological taxonomy remains an integral approach to describing species, the artificial grouping of the genus “*Leptogorgia*” will change soon based on mitogenomic molecular evidence that suggests that genus “*Leptogorgia*” is a polyphyletic taxon with multiples generic geographically restricted lineages (Soler-Hurtado et al. 2017a; Poliseno et al. 2017). The Eastern Pacific “*Leptogorgia*” species are not the oldest nominal assignation and share an immediate common ancestor with the species included to *Eugorgia* and *Pacifigorgia* genera (Soler-Hurtado et al. 2017a; Poliseno et al. 2017).

The discovery of these three new octocoral species was possible because the highest population density of each species was found in relatively isolated marine areas with exceptionally restricted access (Islas Marías Archipelago was a federal penitentiary) or at isolated offshore islands (San Pedro Mártir Island) with access for general public only through touristic trips. However, those locations do not qualify as pristine habitats and they may already be impacted or will be impacted in the near future. The lack of research effort and the small population size of these three new *Leptogorgia* species explain why these species have been overlooked at the Islas Marías Archipelago and oceanic islands of the Gulf of California. The lack of knowledge of *Leptogorgia enrici* sp. nov. is because this species is distributed below 30–40 m deep, often on sandy-pebble sea floors where previous research efforts has been few. Our quantitative, historical, and systematic invertebrate monitoring program has been, so far, focused on fauna from rocky reefs in <20 m depth (Hernández 2014; Ulate et al. 2016; Olvera et al. 2018), thus leaving deeper depths unexplored.

*Leptogorgia martirensis* sp. nov. has been observed and sampled only from rocky reefs from San Pedro Mártir Island (type locality) and San Esteban Island. We observed this species in low density, and only in cavities or caves formed in the island’s rocky reefs. We have been carrying out systematic scuba-diving monitoring during 2008–2019 in at least 50 locations at seven islands located close to San Pedro Mártir, providing strong evidence that, except for San Esteban Island, these islands do not harbor colonies of *L. martirensis* sp. nov. The distribution records of these new species compared with our ecological marine census data at extensive locations along the Pacific coast of Mexico (Breedy and Guzman 2005, 2007; Breedy et al. 2012; Ulate et al. 2016) provide strong evidence that these species are micro-endemic to the Gulf of California. Most islands in the Gulf of California have endemic terrestrial fauna (Álvarez-Castañeda and Ortega-Rubio 2003; Brusca et al. 2005), and this may extend to marine species based on our findings. We show evidence that sea fan micro-endemism also exists in aquatic insular habitats of the Gulf of California, such as Islas Marías Archipelago, San Pedro Mártir and San Esteban Islands, similar to that proposed for the Islas Revillagigedo Archipelago (Olvera et al. 2018).

## Supplementary Material

XML Treatment for Leptogorgia iridis

XML Treatment for Leptogorgia martirensis

XML Treatment for Leptogorgia enrici
